# Introducing *BJGP Open*

**DOI:** 10.3399/bjgpopen17X100749

**Published:** 2017-01-09

**Authors:** Roger Jones, Euan Lawson

**Affiliations:** 1 *BJGP & BJGP Open* Editor and Emeritus Professor of General Practice, King’s College, London, UK; 2 *BJGP* & *BJGP Open* Deputy Editor and Director of Community Studies, Lancaster Medical School, Health Research, Lancaster, UK

Welcome to *BJGP Open*, our new open access, online-only journal of primary care research, practice, and policy. *BJGP Open* has original, high-quality, peer-reviewed primary care research at its core, complemented by reviews, case studies, clinical reports, policy analysis, and the opportunity for discussion and debate.

## Competition for publication

For some time we have been aware that the *BJGP* receives far more high quality original material that it can publish: less than one in four Research articles is accepted, pressure on the non-Research sections of the Journal is intense, and many of the Research articles that we decline are of good scientific quality. The same goes for articles submitted to other sections of the Journal. Primary care everywhere needs to continue to build an evidence base for health care and clinical practice, and to continue to make its political and health policy arguments in all health systems. Peer-reviewed publication is an important means of achieving this. The same is true for education and training in general practice and primary care, and we will welcome original observations and analysis in these fields. *BJGP Open* provides a new opportunity for primary care researchers, clinicians, and policymakers to publish and read a wider scope of original articles of direct relevance to practice, research, teaching, and healthcare delivery.

We hope that we can develop other, more innovative features in our new Journal. For instance, rather than seeing case reports simply as ways of reporting unusual occurrences or as illustrations of well-known principles, we would like to see them interpreted and used more broadly, to illuminate other aspects of care, such as the doctor–patient relationship, continuity and discontinuity, biopsychosocial dimensions of diagnosis and management, new professional roles in primary care, ethical dilemmas, and health policy issues. We hope that, in future issues of *BJGP Open*, the Research section will contain, in addition to original clinical and primary healthcare research, protocols for trials and other planned research studies, and reports of well-conducted educational research. We will also be interested in receiving accounts of novel approaches to delivering primary care services, new directions in primary healthcare policy, and political challenges encountered in primary care, from countries around the globe.

This will all be supplemented by a rapid response eLetters facility, enabling readers to debate important topics with authors and with each other, and through the *BJGP*’s social media channels.

## Open access

Open access publishing is now an established and important part of the medical publication landscape, and *BJGP Open* will be an open-access only journal, which means that Article Processing Charges will be applied and paid by authors, their institutions, or their research funders. We have set these at £1000 for Research articles, and £250 for all other articles in the Journal. These prices, which are substantially lower than most other comparable publications, have been waived and then reduced by 50% for authors of articles accepted in the months leading up to the launch of the Journal, but will be applied in full for articles accepted from 1 March 2017 onwards.

*BJGP Open* will be applying for indexing in PubMed as soon as the first 25 peer-reviewed articles have been published, but — as with all new journals — will need to earn its impact factor. However, as the focus in bibliometrics shifts away from journal impact factors to article-level metrics and other measures of ‘reach’ and impact, we will also focus on the use of alternative metrics — altmetrics — to measure the attention received by individual articles in the journal, and on other measures of their dissemination.

We very much hope that *BJGP Open* will offer new opportunities for primary care publishing, and will develop into an influential global journal of record, debate, and discussion. But, of course, its success will, in the end, depend on you, our readers, authors, and reviewers, who are at the centre of this publishing venture and who I hope will share our enthusiasm for it.

